# Increased Excitability Induced in the Primary Motor Cortex by Transcranial Ultrasound Stimulation

**DOI:** 10.3389/fneur.2018.01007

**Published:** 2018-11-28

**Authors:** Benjamin C. Gibson, Joseph L. Sanguinetti, Bashar W. Badran, Alfred B. Yu, Evan P. Klein, Christopher C. Abbott, Jeffrey T. Hansberger, Vincent P. Clark

**Affiliations:** ^1^Psychology Clinical Neuroscience Center, Department of Psychology, University of New Mexico, Albuquerque, NM, United States; ^2^U.S. Army Research Laboratory, Aberdeen Proving Ground, MD, United States; ^3^Department of Biomedical Engineering, The City College of New York, New York, NY, United States; ^4^Brain Stimulation Laboratory, Department of Psychiatry, Medical University of South Carolina, Charleston, SC, United States; ^5^Department of Psychiatry, University of New Mexico School of Medicine, Albuquerque, NM, United States; ^6^U.S. Army Research Laboratory, Redstone Arsenal, AL, United States; ^7^Department of Neurosciences, University of New Mexico School of Medicine, Albuquerque, NM, United States; ^8^The Mind Research Network & LBERI, Albuquerque, NM, United States

**Keywords:** brain-stimulation, magnetic stimulation, excitability, neuroplasticity, excitation, pulsed ultrasound

## Abstract

**Background:** Transcranial Ultrasound Stimulation (tUS) is an emerging technique that uses ultrasonic waves to noninvasively modulate brain activity. As with other forms of non-invasive brain stimulation (NIBS), tUS may be useful for altering cortical excitability and neuroplasticity for a variety of research and clinical applications. The effects of tUS on cortical excitability are still unclear, and further complications arise from the wide parameter space offered by various types of devices, transducer arrangements, and stimulation protocols. Diagnostic ultrasound imaging devices are safe, commonly available systems that may be useful for tUS. However, the feasibility of modifying brain activity with diagnostic tUS is currently unknown.

**Objective:** We aimed to examine the effects of a commercial diagnostic tUS device using an imaging protocol on cortical excitability. We hypothesized that imaging tUS applied to motor cortex could induce changes in cortical excitability as measured using a transcranial magnetic stimulation (TMS) motor evoked potential (MEP) paradigm.

**Methods:** Forty-three subjects were assigned to receive either verum (*n* = 21) or sham (*n* = 22) diagnostic tUS in a single-blind design. Baseline motor cortex excitability was measured using MEPs elicited by TMS. Diagnostic tUS was subsequently administered to the same cortical area for 2 min, immediately followed by repeated post-stimulation MEPs recorded up to 16 min post-stimulation.

**Results:** Verum tUS increased excitability in the motor cortex (from baseline) by 33.7% immediately following tUS (*p* = 0.009), and 32.4% (*p* = 0.047) 6 min later, with excitability no longer significantly different from baseline by 11 min post-stimulation. By contrast, subjects receiving sham tUS showed no significant changes in MEP amplitude.

**Conclusion:** These findings demonstrate that tUS delivered via a commercially available diagnostic imaging ultrasound system transiently increases excitability in the motor cortex as measured by MEPs. Diagnostic tUS devices are currently used for internal imaging in many health care settings, and the present results suggest that these same devices may also offer a promising tool for noninvasively modulating activity in the central nervous system. Further studies exploring the use of diagnostic imaging devices for neuromodulation are warranted.

## Introduction

Neuroplasticity is fundamental to many neurobehavioral processes, including learning and memory (1). It is believed that neuroplasticity is associated with behavioral changes during normal development, and clinically for post-stroke recovery, traumatic brain injury (2), and adaptation to physical changes in the body (3) among other neural and behavioral changes across the lifespan. It has been suggested that changes in cortical excitability may be related to changes in neuroplasticity (4). Some methods of non-invasive brain stimulation (NIBS) have been found to be effective for inducing changes in brain excitability and, subsequently, neuroplasticity. A number of NIBS techniques have been developed that utilize different forms of energy, including direct and alternating current, magnetic, light, and others (5–8). Each of these techniques have a variety of advantages and disadvantages. Light stimulation, or photobiomodulation, is a promising but as yet little underexplored method of NIBS (9). Transcranial direct current stimulation (tDCS), while inexpensive and associated with minimal side-effects (10), can produce variable results across individuals and time points (11, 12). TMS has a longer history of clinical and experimental application (13), but also comes with more contraindications (14), and is subject to variable effects across individuals (15–17). Recently, ultrasound has received increased interest for NIBS. While the possibility of modulating peripheral nervous system function through ultrasonic stimulation was originally explored in the early twentieth century (18–21), interest subsequently declined, only to be rekindled recently with an emphasis on central nervous system modulation and transcranial ultrasound (tUS) (22–24).

Ultrasonic waves (administered via tUS) are able to pass through the scalp and skull (25–27), where they can safely interact with brain tissue at low intensities (28–30). A number of parameters govern the characteristics of tUS waves, including the fundamental frequency, pulse repetition rate, intensity, duty cycle, and duration of stimulation. Each of these parameters alone, or in combination and in consideration of the precise anatomical regions targeted, has the potential to alter how tUS affects brain activity. However, the exact relationship between tUS parameters and the subsequent effects are not yet fully understood (31–33).

Two common variants of tUS are transcranial focused ultrasound (tFUS), and diagnostic tUS. tFUS typically employs frequencies below 1 MHz, while diagnostic tUS utilizes frequencies ranging from 1 to 15 MHz (34, 35). This distinction is important in tUS, as the human skull is believed to attenuate the energy of higher frequency US more greatly than lower frequencies (36–38), with the degree of energy absorption and wave aberration varying across individuals (26, 39). Diagnostic tUS can be used to image brain tissue through the skull (26, 40–43), demonstrating that energy can be successfully passed into and out of the skull and brain at higher frequencies than those typically used in tFUS applications. Whether or not the amount of energy passed into the skull with diagnostic tUS is sufficient to produce neurophysiological effects is a question that has not been examined previously.

A growing body of literature has formed around the use of various forms of tUS in small mammals (35, 44–47) and non-human primates (48, 49), paving the way for research with human subjects (50). In separate studies, tFUS applied to the human somatosensory cortex improved performance on a tactile discrimination task (51), and elicited transient tactile sensations in the contralateral hands and fingers (52). Diagnostic tUS has also been applied for the purpose of neuromodulation. Administering 8 MHz diagnostic tUS over the temporal window, Hameroff and colleagues reported that 15 s of stimulation acutely improved subjective mood (53). While similar, longer term effects following tUS stimulation have been observed (54, 55), the brain processes underlying these changes are yet to be fully elucidated (56).

Here we examined whether tUS administered using a diagnostic ultrasound system modulates cortical excitability in healthy adults by using motor evoked potentials (MEPs) induced by transcranial magnetic stimulation (TMS) (57, 58).

## Methods

### Subjects

Sixty-six healthy participants (42 female) participated in this randomized, single-blind study exploring the effects of tUS on cortical excitability. Individuals were required to pass a tUS and TMS screening form which included the following: Right-handed, age 18–45, no personal or family history of seizure, mood, or cardiovascular disorders, no facial or ear pain, no recent ear trauma, no metal implants including pacemakers, not pregnant, no dependence on alcohol or recent illicit drug use, and no use of any pharmacological agents known to produce significant changes in CNS function or increase seizure risk. A between-subjects design was chosen because, while TMS elicited MEPs have been shown to be reliable across sessions (59, 60), the intra-individual reliability of other forms of NIBs across sessions is still debated (61–64). This is especially a concern for forms of NIBS that are neuromodulators, like TUS, where daily changes in endogenous brain activity can have a large impact on the outcome of stimulation (6, 65, 66).

All experimental procedures were approved by Chesapeake IRB and the U.S. Army Research Laboratory's Human Research Protection Program.

### Experimental overview

**Figure 1:**
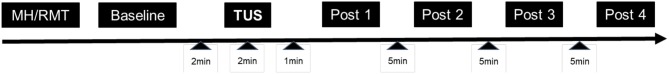
Study Design. The experimental visit lasted between 70 and 100 min. MH, acquisition of the motor hotspot; RMT, acquisition of resting motor threshold.

Participants were seated in a reclining chair, informed about the study, and consented. To check for changes in subjective psychological state over the length of the protocol, subjects then completed a brief mood questionnaire that asked them to endorse 10 statements using a 6-point (0–5) Likert scale (Table 1). This same questionnaire was administered again at the conclusion of the study. Following measurement of baseline cortical excitability, subjects received either verum or sham tUS to their motor cortex for 2 min. Cortical excitability was measured immediately after stimulation at 1 min, and at 5 min intervals up to 16 min post-stimulation (Figure 1). Sham control was accomplished through application of the freeze function on the machine prior to transducer application, as has been employed in other studies using diagnostic tUS (53, 67). Subjects completed sensation questionnaires following acquisition of the motor threshold and again following tUS. These asked subjects to separately rate the degree of itching, heat/burning, and tingling on a 0–10 scale.

**Table 1:** Questionnaire administered prior to and after stimulation to probe possible changes in subject-reported psychological state.

**Mood questionnaire items**
1) I feel nervous
2) I feel excited
3) I feel tired or fatigued
4) I feel confused or disoriented
5) I feel sad or down
6) I feel tense or frustrated
7) I feel dizzy or light-headed
8) I feel nauseous
9) Physically, I feel pain or discomfort
10) I feel unable to concentrate or pay attention

### Cortical excitability recording using TMS-induced MEPs

TMS-induced MEPs (57, 58) were administered using a neuronavigation-assisted eXemia TMS system (Nextstim Ltd., Helsinki, Finland) with a 70 mm figure of eight coil and NBS software (version 3.2.0). Electromyography (EMG) was recorded from disposable Ambu Neuroline 720 electrodes attached to the abductor pollicis brevis and opponens pollicis muscles of the right hand with the reference electrode attached to the base of the extensor digitorum tendon of the right-hand middle finger. This enables recording of MEPs elicited from contraction of the thumb.

Subjects were instructed to rest their hand on a pillow in a relaxed position, where it remained for the duration of the study. MEPs are highly variable (68, 69), less variable resting motor thresholds (RMT) were determined for each subject (70). Subject's RMT was determined through adaptive parametric estimation via sequential testing (PEST) procedure and software (TMS Motor Threshold Assessment Tool, MTAT 2.0, (http://www.clinicalresearcher.org/software.html), which reliably determines the motor threshold (71–73). Prior to baseline, TMS power output was set at 110% of the power associated with an individual subject's RMT, where it remained for the duration of the experiment. In a given subject, if the PEST procedure found that acquisition of the RMT required a TMS power output that exceeded the total possible power output of the TMS system, then the experimental session was discontinued and the subject was regarded as not having consistently measurable MEPs.

The motor hotspot associated with abductor pollicis brevis activation was identified through a combination of visual inspection and EMG, with the area most consistently eliciting MEPs above 1 mV coupled with isolated thumb movements being selected for the RMT procedure, using similar methodology as Nitsche and Paulus (58). Stimulated areas of the motor cortex were tracked and mapped via neuronavigated TMS through the eXemia system. Neuronavigated TMS has demonstrated a higher probability of finding consistent MEPs compared to referencing external landmarks (74–77), as it allows the experimenter to maintain the location of the motor hotspot as well as the ideal coil orientation for an individual subject (78, 79).

Baseline excitability was measured through a series of 10 single TMS pulses delivered an average of 4 s apart. Following tUS, 4 additional blocks of 10 single TMS pulses were performed: 1 min after tUS application and then 3 more blocks of 10 pulses each separated by 5 min intervals.

### Ultrasound stimulation

We used a Phillips CX50 Diagnostic Imaging Ultrasound System, with a Phillips S5-1 broadband plane sector transducer array. This transducer has 80 piezoelectric elements, an aperture of 20.3 cm, and a frequency range of 1–5 MHz. The system was set in HGen, B-mode with harmonics on and a focal depth of 10 cm. The waveform generated by this transducer occurs in a plane wave where the energy deposited is homogenous across the field of view. The central frequency was 2.32 MHz, which represents the median frequency emitted by the transducer, with the absolute range of frequencies normally distributed between the limits of 1.53 and 3.13 MHz (80). SonicEaze ultrasound conductive gel was used to create an acoustic medium when applying the transducer to the scalp. To ensure fidelity to the previously identified hot spot, neuronavigation was again employed for tUS transducer placement. In order to measure maximum acoustic output, a hydrophone (HNR 500, Onda Corporation, Sunnyvale, CA, United States) calibrated 1 month prior to testing was used. The peak negative pressure associated with our transducer settings was 1.02 MPa as measured in free, degassed water with a manual stage.

### Statistical analysis

To explicate excitability changes associated with tUS, data was analyzed in SPSS using a between-subjects repeated measure ANOVA with 2 conditions, verum and sham, and 5 time points as described above. Student's *t*-tests (independent samples, two-tailed, *p* < 0.05) were then performed to test between-group differences at each post-stimulation time point. Individual subject MEPs were averaged across the 10 stimuli given per block. Individual TMS pulses that elicited an MEP amplitude of 0 were discarded and not counted in the 10 MEP average. The researcher performing the analysis was not blind to the experimental groups; however, there were no subjective steps involved in the MEP analysis that could be unduly influenced by unblinding. Due to the limited extant literature utilizing higher frequency tUS, no *a-priori* hypotheses were made about the direction of any possible neuromodulation effects.

## Results

### Subjects

**Figure 2:**
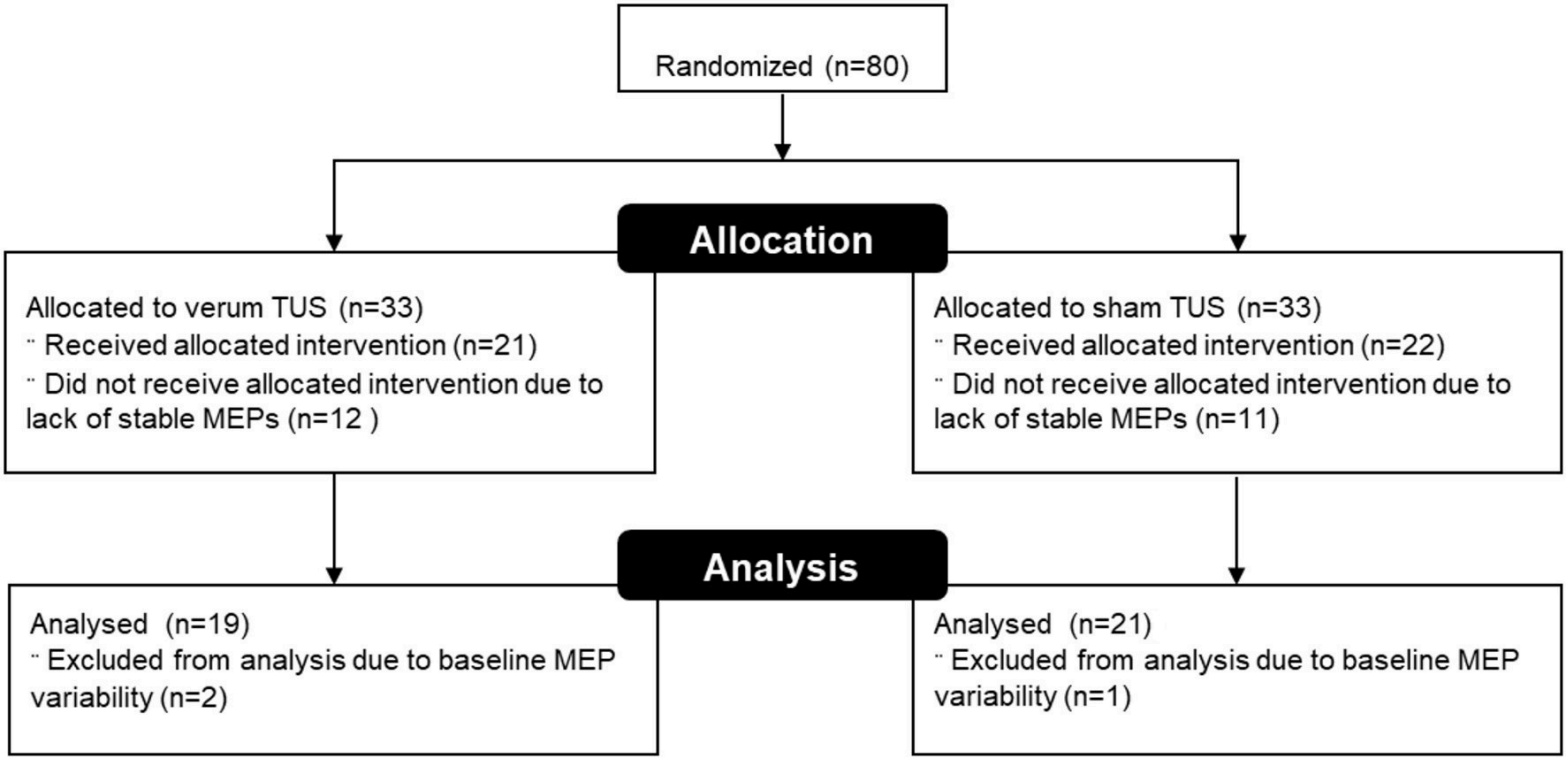
Flow chart showing subject randomization and exclusion.

Eighty subjects were assigned to the current study after passing screening. Fourteen of these (17.5%) canceled due to scheduling conflicts or could not participate to due illness or other issues, leaving 66 subjects that were enrolled and consented. Measurable MEPs could not be obtained in 23 subjects (35%), and these were excluded from further analysis. We collected MEP data from the remaining 43 subjects, 21 who received verum tUS and 22 who received placebo tUS. The mean MEP averages at baseline were 0.932 mV for the verum group and 0.849 mV for sham (see Figure 2). This difference in baseline means between groups was not significant (*p* = 0.55). Subjects with baseline intra-block variability >1 standard deviation (> 0.812 mV) above the average variability for all baseline trials (*N* = 420) were excluded, which led to 2 additional exclusions in the verum group and 1 in the sham group. No subjects had more than 2 MEPs of 0 within a single block. A total of 40 subjects were used in the analysis, with 19 in verum (11 females) and 21 in sham (14 females). The mean age was 20.58 (*SD* = 1.5) and 22.05 (*SD* = 5.0) for verum and sham, respectively. The median age in the verum group was 21, with a range of 19 to 23. In the sham group, the median age was 20, with a range of 18 to 38. There were no significant differences between these final groups in gender or age composition (*p* > 0.05). Additionally, the difference in RMT as percentage of TMS power output between groups was not significant (*p* > 0.05; Table 2).

**Table 2:** One hundred and ten percent resting motor thersold as percentage of TMS machine output.

		**110% RMT**
	***n***	***M (SD)***	**Range**
Sham	21	78.6(10.84)	51–94
Male	7	81.71(10.14)	66–94
Female	14	77.29(11.24)	51–93
Verum	19	72.05(11.03)	51–94
Male	8	70.38(12.33)	56–94
Female	11	73.27(10.42)	51–88

### Safety

The most commonly reported sensation was tingling, both before baseline measurement (*M* = 1.44, *SD* = 1.58), and after tUS (*M* = 1.32, *SD* = 1.65). No significant differences were found between these pre- and post-stimulation measures for any of the sensation questions regarding itching, heat/burning, and tingling, nor were there any significant differences between verum and sham groups at either time point.

The mechanical index during tUS observed during hydrophone measurement was 0.67, well below the mechanical index limit of 1.9 recommended by the Food and Drug Administration (81). The thermal index reading of 2.6 was also well within established safety parameters (28), where tissue can safely be exposed to similar temperatures for up to 100 min (82). The low duty cycle of our device, < 1%, led to an I_sppa_ of 34.96 W/cm^2^ and an I_spta_ of 132.85 mW/cm^2^ before transcutaneous and bone transmission (i.e., in free water), well below recommended limits of 720 mW/cm^2^.

As an additional safety measure, we assessed the possibility of motor cortex stimulation eliciting acute changes in subject mood with a brief questionnaire. Across both groups, paired samples *t*-tests indicated significant changes in 2 items over time. On a scale from 0 to 6, subjects reported feeling less nervous (*M* = 0.96, *SD* = 1.11) and less excited (*M* = 2.44, *SD* = 1.56) after completion of the study (nervous *M* = 0.32, *SD* = 0.63, *p* = 0.003; excited *M* = 1.72, *SD* = 1.48, *p* = 0.011). No significant differences between groups were found either before or after stimulation for any of these measures.

### Effects on cortical excitability

**Figure 3:**
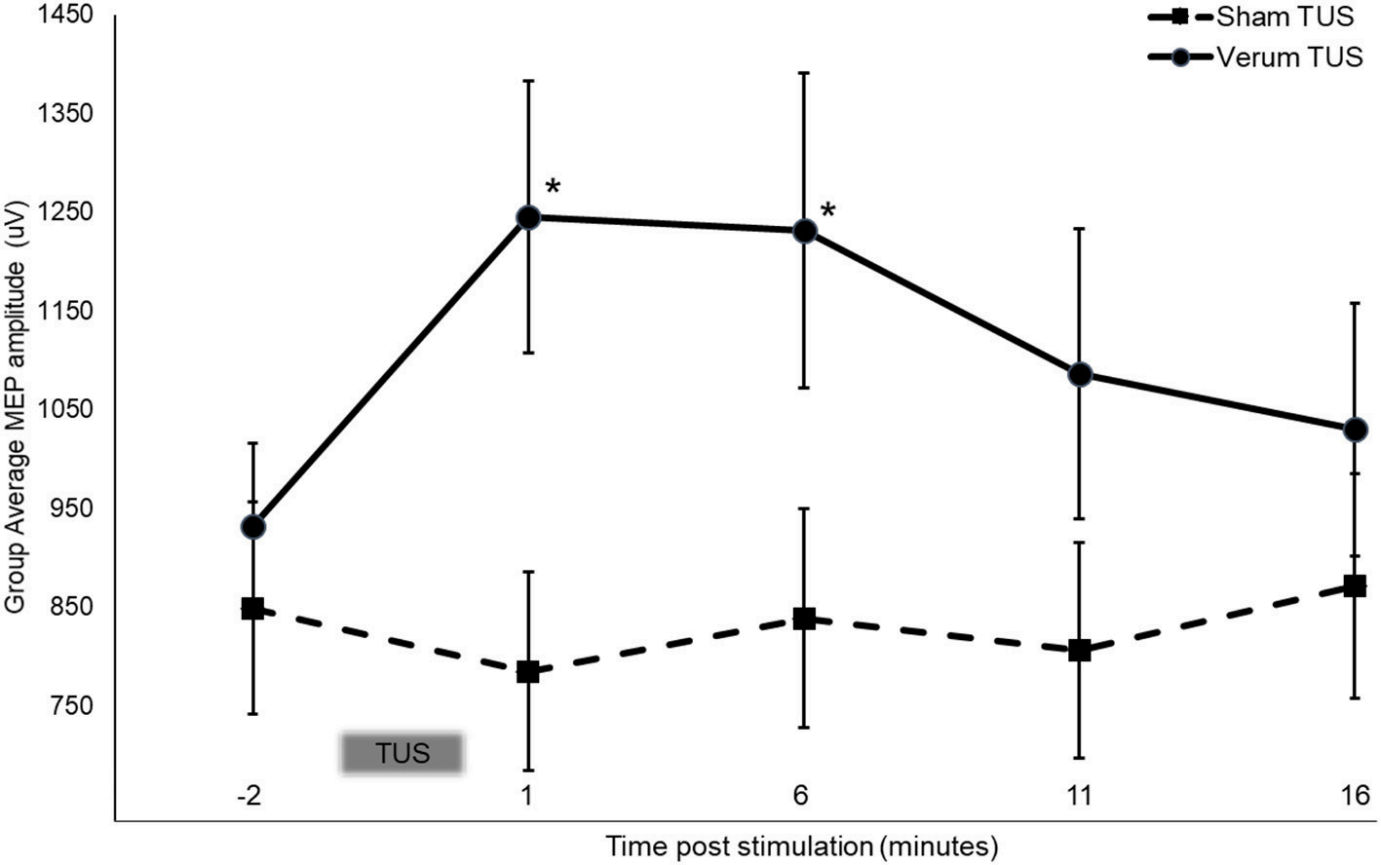
Stimulation dependent changes in MEP amplitude at baseline and following 2 min of tUS. Asterisks indicate significant between group differences (two-tailed *t*-test, independent samples, *P* < 0.05) Error bars = ±SE.

Mauchly's test was significant, χ^2^ (9) = 18.51, *p* = 0.03, indicating unequal variances between verum and sham groups, therefore a Greenhouse-Geisser correction was used (ε = 0.851). There was a significant interaction between condition and time point [ANOVA, *F*_(3.404, 129.349)_ = 3.501, *p* = 0.014, ω^2^ = 0.059]. For subjects that received verum tUS, stimulation produced an average 33.7% (*SD* = 0.457 mV) increase in average MEP amplitude 1 min post stimulation (post measure 1) that declined slightly to 32.2% (*SD* = 0.511 mV) over baseline 6 min post stimulation (post measure 2; Figure 3). This contrasted with sham subjects whose average MEP amplitude was 7.6% (*SD* = 0.187 mV) smaller than baseline at post measure 1 and 1.2% (*SD* = 0.290 mV) smaller at post measure 2. Follow up comparisons revealed a significant difference between sham (*M* = 0.785 mV, *SD* = 0.460 mV) and verum tUS (*M* = 1.246 mV, *SD* = 0.600 mV) at 1 min post-stimulation (post measure 1), *t* (38) = −2.750, *p* = 0.009; and at 6 min post-stimulation (post measure 2), verum tUS (*M* = 1. 232 mV, *SD* = 0.694 mV), sham tUS (*M* = 0.839 mV, *SD* = 0.510 mV), *t* (38) = −2.054, *p* = 0.047. Effect sizes for between-groups comparison were calculated using a pre-post control technique that accounts for groups of unequal sample size (83). At post measure 1 the observed effect size was *d* = 0.86, and *d* = 0.71 for post measure 2. No significant differences in MEP amplitude were found between groups for post-measures 3 (*p* = 0.129) and 4 (*p* = 0.359), collected at 11 and 16 min post stimulation.

## Discussion

This study demonstrated effects of tUS on MEP amplitude, amounting to a 33.7% increase in average MEP amplitude 1 min and a 32.2% increase 6 min after stimulation. Thereafter, MEP amplitude decreased toward baseline, and was not significantly different than sham for the remainder of testing times. Control subjects' average MEP amplitude was not significantly different than baseline at any post-stimulation timepoint. The 2 min duration of tUS used here produces neurophysiological effects that are limited in time, in this case approximately 4 times the duration of stimulation. Studies using other forms of NIBS, such as tDCS and TMS, have observed a similar relationship (57, 84–x^86^), and have found that a longer duration of stimulation led to longer effects. Results using these other modalities suggest that the duration of tUS effects might be controlled in part by varying the duration of stimulation.

The length of tUS induced changes we observed corresponds with previous research, where suppression of visual evoked potentials and somatosensory evoked potentials following tFUS lasted between 5 and 10 min in (54, 87). Importantly, neither of these studies found evidence of tUS induced tissue damage in histological analyses. In humans, Hameroff et al. found mood effects that persisted up to 40 min after stimulation (53). While we did not find similar mood changes following stimulation, our questionnaire was designed to be a brief status check on subject well-being and not a nuanced accounting of mood effects, so subtle changes in mood may have been missed. A possible alternative explanation might be that tUS effects are specific to the location of stimulation, and that stimulation of the temporal lobe produces mood effects, while stimulation of the motor cortex does not.

Our study is also one of many to have found excitatory effects from tUS. At the neuronal level, ultrasound stimulation has directly evoked electrical responses from extracted cells (23, 88), opened voltage-gated sodium and calcium channels (24), and increased the concentration of excitatory neurotransmitters (46, 89). In small mammals, *in vivo* tUS excitation of the motor cortex has also often been observed, accompanied by increases in BOLD activation, EMG amplitude, and evoked movement (44, 45, 87, 90). Beyond the motor cortex excitation has been measured by visual evoked potentials (91, 92), EMG (93), directly evoked movement (35, 94–96), and increased glucose uptake (97). Similar excitation has been found in humans as well, measured by increased somatosensory evoked potentials (52), increases in BOLD activation (93, 98), and increases in the volume of activated cortical tissue in the motor cortex (99).

Our findings of increased MEP amplitude with tUS contrast with recent work that investigated the effect of transcranial tFUS on MEPs where a reduction in MEP amplitude was demonstrated (100). There are a number of differences between these studies that might account for the difference in response polarity observed. The precise tUS system, transducer types, and the tUS protocols used were all different between studies. TFUS and tUS using a diagnostic ultrasound system potentially penetrate to different cortical depths, with higher frequency diagnostic tUS possibly affecting more dorsal cortical tissue nearer to the scalp, and tFUS affecting deeper tissues that are out of the direct reach of subsequent TMS stimulation (100, 101). The differences in findings might also be due to contrasting methodologies between the present study and others, where applying tUS simultaneously with TMS leads to inhibitory (100), or null effects (67), whereas serial application as used here leads to excitatory effects.

While supported theories exist for the effects of tUS (32, 102, 103), researchers are still coming to grips with how tUS effects the brain above the level of individual neurons. Further complication comes from trying to parse how the numerous parameters of tUS interact with each other and with the stimulated medium. Another difference between our study and that of Legon and colleagues is the volume of tissue stimulated, a factor that might be crucial in interpreting the effects of tUS generally. Holding all other parameters constant, unfocused transducers impact upon more brain area than focused transducers, and analogously, each decrease in frequency within a focused transducer serves to increase the stimulated area. The greater brain volume affected by unfocused stimulation, as compared to focused stimulation, might thus be conceptually similar to the increased brain volume that is affected by lowering the frequency of focused ultrasound. In both cases, the sheer volume of brain tissue involved might be more important than the total energy delivered. If acoustic force was the most important parameter for induced effects, higher frequencies would generally equate with stronger effects; however, lower frequencies, and thus larger stimulated areas, have been found to be more likely to get a response (35, 44, 45). TFUS has also been shown to require greater energies to elicit an excitatory motor response, I_sppa_ of 12.6 W/cm^2^, than unfocused tUS, I_sppa_ of 0.23 W/cm^2^ (45, 87, 104). The same might hold true for human studies, where higher frequencies, >0.500 MHz, lead to inhibition (51, 100, 105), and lower frequencies, < 0.350 MHz (52, 93, 106), or unfocused stimulation (53) induce excitation. The present study used a center frequency of 2.32 MHz, further suggesting that frequency may not be an important parameter in comparing excitatory and inhibitory effects, and that the volume of tissue affected may be the critical parameter. Furthermore, given our use of an unfocused transducer operating a relatively high fundamental frequency, acoustic energy reaching the brain may have been distributed across an even larger area due to diffuse refection occurring within the diploë layer of the skull (31, 107).

A limitation of this study was the use of a single blind experimental design. While the results of any single blind study must be interpreted with caution, no significant differences in outcome measures have been observed in prior studies from our laboratory comparing single- vs. double-blind NIBS on objective outcome measures (108). Two other points also help to mitigate the potential impact of the use of a single-blind design here. First, studies have shown that objective measures, such as the MEPs collected here, are less sensitive to expectancy effects compared with more subjective measures (109, 110). Second, and most importantly, a chi-square test was conducted and no significant relationship was found between assigned condition and condition guessed by the subject at the conclusion of the experiment, χ^2^ (1, *N* = 40) = 1.50, *p* = 0.22. In addition, a greater percentage of sham subjects, 71.4%, reported that they believed they were in the verum condition, compared to 52.6% of actual verum subjects.

Another limitation was the relatively high number of participants excluded from the study. This was due in part to the power output of our TMS system, where we found that the average RMT was 68.7% of TMS power output. Thus, for excluded participants, our baseline TMS power of 110% RMT exceeded the total possible power output of the TMS machine, and so could not be performed for those participants. It should also be noted that the average age in our sample was 21.35. Replication with older subjects is thus needed, as age has been previously shown to impact NIBS mediated plasticity (111). Such replication is essential for possible clinical application. Other forms of NIBS have been explored as possible therapies for movement disorders (112–115), and given the observed tUS-induced changes in the primary motor cortex, this might be a productive avenue for future tUS research.

## Conclusion

Our results demonstrate that tUS produced by a diagnostic imaging ultrasound system increased short-term cortical excitability in the motor cortex. This suggests that diagnostic tUS systems may be used as a neuromodulatory tool to alter the activity of the primary motor cortex. Following similar evidence demonstrating the effect of tDCS on excitability of the primary motor cortex as measured by TMS-evoked MEPs (57, 58), future research should determine how the observed tUS effects translate to other cortical areas and other measures of neuromodulation. By contrast to tES and TMS, tUS offers the advantage of greater anatomical precision and also greater depth without significant effects in more superficial regions, which together may allow for greater precision and rigor in this research, and ultimately may offer improved methods of treatment. Our finding of excitatory effects from tUS contrasts with a recent report of inhibitory effects (100), suggesting the potential for a wide dynamic range in cortical excitability using tUS. Ultrasound imaging has been used for many years and has an excellent safety record. The present results warrant further research into the use of diagnostic imaging ultrasound to modulate cortical excitability and neuroplasticity beyond the motor cortex, as well as the development of new clinical applications for this technology. If further study and development confirm that diagnostic imaging ultrasound is effective for producing neuromodulation, and given that diagnostic imaging ultrasound devices are found in many clinical settings worldwide alongside technicians trained in their use, this could potentially make neuromodulatory tUS highly accessible to clinical and research communities.

## Ethics statement

This study was carried out in accordance with the recommendations of Chesapeake IRB and the U.S. Army Research Laboratory's Human Research Protection Program, with written informed consent from all subjects in accordance with the Declaration of Helsinki.

## Author contributions

Conceptualization JS and VC; methodology BB, BG, JS, and VC; software JS and BG; formal analysis BG and JS; investigation BG, JS, and EK; resources AY, JH, and VC; writing—original draft preparation, BG and JS; writing—review and editing, all authors; supervision, JS and VC; project administration, VC; funding acquisition, VC.

### Conflict of interest statement

JS is Chief Scientific Officer of Alchemas, Inc. (Redwood City, CA), a research company investigating focused ultrasound neuromodulation. The remaining authors declare that the research was conducted in the absence of any commercial or financial relationships that could be construed as a potential conflict of interest.
